# Influence of Protein Intake from Haem and Non-haem Animals and Plant Origin on Inflammatory Biomarkers among Apparently-healthy Adults in Greece

**DOI:** 10.3329/jhpn.v31i4.19992

**Published:** 2013-12

**Authors:** Natalia G. Vallianou, Vassiliki P. Bountziouka, Ekavi Georgousopoulou, Angelos A. Evangelopoulos, Maria S. Bonou, Evangelos D. Vogiatzakis, John D. Barbetseas, Peter C. Avgerinos, Demosthenes B. Panagiotakos

**Affiliations:** ^1^Polykliniki General Hospital, Athens, Greece; ^2^Department of Nutrition and Dietetics, Harokopio University of Athens, Athens, Greece

**Keywords:** Diet, Haematocrit, Inflammation, Protein intake, Greece

## Abstract

Intake of different types of protein may be associated with differences in biomarkers among various populations. This work investigated the influence of protein intake from haem and non-haem animals as well as protein from plants on haematological and biochemical parameters in inflammation among apparently-healthy adults living in Greece, a Mediterranean country. Four hundred and ninety apparently-healthy subjects (46±16 years, 40% men), who consecutively visited Polykliniki General Hospital for routine examinations, voluntarily agreed to participate in the study (participation rate 85%). Demographic, anthropometric and lifestyle characteristics were recorded. Participants completed a valid, semi-quantitative food frequency questionnaire. Protein intake was classified into three sources: protein from haem animals, protein from non-haem animals, and protein from plant origin. Fasting blood samples were taken from all participants; uric acid, creatinine, lipids, cystatin C, haptoglobin, haemoglobin, haematocrit, iron, ferritin, white blood cells, monocytes, platelets, and C-reactive protein were measured. Protein intake from only haem animals was associated with increased haemoglobin and haematocrit levels (p<0.05) whereas intake of protein from non-haem animals and plant origin was not associated with the investigated haematological and biochemical markers of low-grade chronic inflammation when lifestyle factors and overall dietary habits were taken into account. Intake of protein from only haem animals seems to be consistently associated with haematological markers. The confounding role of dietary habits and lifestyle variables on the tested parameters deserves further attention in future research.

## INTRODUCTION

Protein intake contributes to 15-20% of the total daily energy consumption for adult people, although intake of the proportion of protein subtypes, i.e. haem and non-haem animals, and plants, varies among individuals. The effects of intake of the various types of protein on human health have long been studied but its association with cardiometabolic diseases is still conflicting. For example, intake of vegetarian-based diets that are low in haem protein and high in plant-originated protein has been associated with lower risk of cardiovascular diseases and some types of cancer (1). The Mediterranean people, particularly Greek, who consumed plant-oriented diets rich in vegetables, fruits, and cereals early after the Second World War, had lower rates of mortality from cardiovascular diseases in the 1970s and 1980s compared to many other parts of the world (2). Metabolic studies have shown that protein intake from haem animals may significantly affect haematocrit status, platelet count, and levels of various inflammation markers due to the fact that it contains more iron than does non-haem protein (3-10). Iron balance is precisely regulated mainly through changes in the amount of iron absorbed from the gastrointestinal tract that is determined by the iron content in the meal, the chemical form of the iron, the iron status of the individual, and composition of the ingested food (5-7). Primarily plant-based diets were considered to have low iron bioavailability because of their almost exclusively non-haem iron content, combined with reduced or negligible amounts of dietary enhancers of non-haem iron absorption and large amounts of dietary inhibitors found in staples, such as beans, cereals, beverages, and spices (8,9). However, in the context of a Western-type diet, high bioavailability of iron was observed, mainly because of the intake of haem animal protein (10,11). Recently, iron has been suggested to promote atherosclerosis due to its remarkable capacity of free radical generation; however, the molecule and the biomarkers relating to this are under extensive study (3). Despite the aforementioned reports, there is a lack of consistent information about the effect of the origins of protein on haematological and biochemical features relating to inflammation process, especially among healthy people.

The aim of the present work was to examine whether the consumption of different types of protein (i.e. protein from haem and non-haem animals and that from plant origin) is associated with platelets count, haemoglobin, haematocrit, ferritin, blood lipids, renal function markers as well as various inflammation markers directly related to cardiometabolic risk, like cystatin C (12), C-reactive protein (13), white blood cell count (14), uric acid (15), haptoglobin (16) among apparently-healthy adults.

## MATERIALS AND METHODS

### Participants

Between April 2009 and January 2010, a total of 490 consecutive Caucasian adults from 577 patients who visited the Polykliniki General Hospital for an annual health check-up were informed about the aim of the study, and they agreed to participate providing written informed consent. Individuals with history of cancer or recent viral infection (i.e. urinary, respiratory, and dental), abnormal fever during the last month, or use of antibiotics, were not included in the study. The retrieved data were confidential, and the study followed the ethical considerations provided by the World Medical Association (52nd WMA General Assembly, Edinburgh, Scotland, October 2000). Moreover, the Institutional Review Board approved the design, procedures, and aims of the study (GA 23/14.05.2009). The sample-size was adequate to achieve statistical power of 80% or higher for the evaluation of 10% change (two-sided) in standardized beta-coefficients of regression models regarding protein intake and the investigated biomarkers at 5% significance level.

### Anthropometric, clinical and lifestyle measurements

Waist-circumference and height (without shoes) were measured to the nearest 0.5 cm, and weight (without shoes and in light underwear) was measured with a lever balance to the nearest 100 g. Body mass index (BMI) was calculated as weight in kg divided by the square of standing height in metres (m^2^). Blood pressure was measured by the same physician, using a standard mercury sphygmomanometer on the right arm of the seated subject. Blood pressure measurements were done twice on the right arm with a time gap of 15 minutes, and the mean value was used. Hypertension was defined as a systolic blood pressure ≥140 mmHg and/or a diastolic blood pressure ≥90 mmHg, or the use of antihypertensive drugs. Hypercholesterolaemia was defined as serum cholesterol >200 mg/dL or from the use of cholesterol-lowering drugs. Diabetes was assessed by self-report, medication-use, or a positive diagnosis from the level of fasting blood glucose (≥7.0 mmol/L) (17).

With respect to physical activity status, participants were asked to fill a 10-grade scale regarding the frequency and intensity of their activities (1 denotes sedentary lifestyle and 10 means daily hard activities for at least 30 minutes). Current cigarette smoking was also recorded. Dietary intake was assessed by a valid semi-quantitative 76-item food frequency questionnaire (FFQ) (18). Alcohol consumption was assessed as the number of drinks per week. Food items were classified according to their content of protein in three groups: non-haem (i.e. dairy products and egg), haem (i.e. meat and meat products, fish, and seafood), and protein from plant origin (i.e. pulses). Protein intake, according to this classification, was categorized in tertiles, indicating low, moderate and high protein intake and was energy-adjusted using the energy density model. Furthermore, overall assessment of dietary habits in terms of adherence to the Mediterranean dietary pattern was evaluated through the MedDietScore (range 0-55) (19,20). Higher values in the score indicate greater adherence to this pattern and, consequently, healthier dietary habits. Finally, education status, as a proxy of social status, was measured in years of schooling.

### Laboratory analyses

Venipuncture was performed for each participant after a 12-hour fasting period by applying a natural latex rubber strap and using a 20-mL syringe. Blood was immediately transferred to two tubes without anticoagulant (Greiner Vacuette, Cat. No. 455071) for serum separation and one tube containing K3EDTA (Greiner Vacuetter, Cat. No. 454003). Total cholesterol, HDL-cholesterol, and triglycerides were determined by enzymatic colorimetric tests. Serum creatinine was determined via a kinetic colorimetric assay based on the reaction of creatinine with picric acid in alkaline solution. Cystatin C, hsCRP, ferritin, and haptoglobin were determined via immunoturbidimetric assays. In particular, for the determination of cystatin C, human cystatin C in serum agglutinates with latex particles coated with anticystatin C antibodies and the aggregate is determined turbidimetrically at 546 nm. Reproducibility in the laboratory has been determined using human samples and controls in an internal protocol. For the abovementioned tests, within-run and between-day coefficients of variation (CV) were less than 7%. Control recovery for all tests was very close to the recommended target values (TV±5%). Accuracy of results is further supported by participation in suitable external quality assurance programmes (ESEAP). All measurements were performed on a Roche/Modular Analytics analyzer. Reagents, calibrators, controls, and consumables were purchased from the same supplier (Roche Diagnostics GmbH, Sandhofer Strasse 116, D-68305 Mannheim, Germany). Total and differential leukocyte counts were determined in an automated haematology analyzer (Advia 2120, Siemens). Accuracy of results was further supported by participation in suitable external quality assurance programmes (Hellenic Association of Hematology).

### Statistical analysis

Results are presented as mean±standard deviation (SD) for the normally-distributed continuous variables (i.e. age, physical activity, MedDietScore, BMI, uric acid, creatinine, total cholesterol, HDL-cholesterol, LDL-cholesterol, cystatin C, haptoglobin, haemoglobin, haematocrit, white blood cells, monocytes, and platelets); as median (P25-P75) for the skewed ones (i.e. years of schooling, urea, triglycerides, iron, C-reactive protein, ferritin); and as frequencies (%) for the categorical variables (i.e. sex, smoking, presence of obesity, hypertension, hypercholesterolaemia, and diabetes). Normality was tested using the P-P plots for the continuous variables. Pearson's chi-square test, one-way ANOVA, and the Kruskal-Wallis H test were used in evaluating differences in various sociodemographic, lifestyle and clinical characteristics and blood levels of biomarkers according to protein intake. Furthermore, linear regression models were used in evaluating the association of protein intake from various sources with levels of biomarkers, adjusted for age, gender, BMI, physical activity, smoking, alcohol drinking, and MedDietScore. Collinearity between the independent covariates was tested using the VIF criterion (values >4 indicates collinearity). Normality of the residuals was tested using the P-P plots; linearity of the covariates was tested using the scatter plot of the residuals vs the predicted values; and serial dependency of the dependent variables was tested using the Durbin-Watson test. Statistical significance was defined at p<0.05. All statistical analyses were performed using the SPSS (version 18.0) (SPSS Inc., Chicago, IL, USA).

## RESULTS

In Table 1, the distribution of the sociodemographic, lifestyle and clinical characteristics of participants is presented according to the type of protein intake. The prevalence of hypercholesterolaemia and hypertension was higher among those categorized in the lowest tertile of haem protein intake (p<0.05). No other significant associations were observed regarding medical history or BMI and type of protein consumed (p>0.40). Smoking habit decreased as protein intake from non-haem animals and plants increased (p<0.03) while the level of adherence to the Mediterranean diet increased as the level of protein intake from non-haem animal and plant origin increased (p<0.01). Finally, participants in the upper tertile of protein intake from haem animals had higher educational level and were younger compared to the lowest tertile (p<0.001) (Table 1).

With respect to levels of biomarkers, unadjusted analyses showed differences between consumption of various types of protein and cystatin C, haemoglobin, haematocrit, triglycerides, iron levels, and blood platelets count. Specifically, blood-level concentration of cystatin C decreased as the frequency of haem animal protein increased (p<0.001) while haemoglobin and haematocrit levels increased (p<0.001). **A** decreasing trend was observed regarding triglycerides, iron, haemoglobin, and haematocrit as the frequency of consumption of non-haem animal protein increased (p<0.05 for all). Also, blood platelets count reduced as protein intake from plant origin increased (p=0.02) (Table 2).

**Table 1.** Sociodemographic, lifestyle and clinical descriptive characteristics of participants, with respect to the type of protein intake (Results are presented as relative frequencies, mean±SD, or P50 (P25-P75) (N=490)

Characteristics	Animal								protein			
	Haem				Non-haem							
	Low	Moderate	High	P†	Low	Moderate	High	p†	Low	Moderate	High	p†
	n=164	n=163	n=163		n=164	n=163	n=163		n=164	n=163	n=163	
Males, %	32	40	48	0.01	51	41	27	<0.001	37	44	38	0.40
Age of participants, years	50±17	48±16	41±14	<0.001	45±16	46±16	47±16	0.72	46±17	45±15	48±16	0.32
Years of schooling	12 (10-16)	14 (12-16)	15 (12-17)	<0.001	14 (12-16)	14 (12-16)	14 (12-16)	0.66	13 (11-16)	14 (12-16)	14 (12-16)	0.32
Physical activity, 1-10	5.4±2.3	5.7±2.2	5.6±2.2	0.62	5.3±2.6	5.6±2.2	5.8±202	0.24	5.6±2.2	5.3±2.4	5.8±2.1	0.14
Smoking, %	27	36	37	0.10	43	28	28	0.006	40	34	26	0.03
Mediterranean dietary score, 0-55	30±4.1	30±4.2	29±4.3	0.44	29±4.2	30±4.0	30±4.2	0.007	27±4	30±3.2	32±3.6	<0.007
Body mass index, kg/m2	26±5.1	27±4.4	26±5.2	0.66	26±4.7	27±4.7	27±5.3	0.94	26±4.5	27±5.0	27±5.2	0.31
Obesity, %	24	22	25	0.53	20	26	24	0.37	20	24	26	0.62
History of hypertension, %	33	27	21	0.05	27	29	24	0.60	25	28	28	0.79
History of hypercholestero-laemia, %	63	56	47	0.01	54	56	56	0.95	54	53	59	0.48
History of diabetes, %	7.9	7.4	6.1	0.82	6.7	7.3	7.5	0.96	7.3	5.6	8.5	0.58

†p values derived from chi-square test for the categorical variables (i.e. sex, smoking, presence of obesity, hypertension, hypercholesterolaemia, and diabetes), one-way ANOVA for the normally-distributed variables (i.e. age, physical activity, Mediterranean Diet Score, and body mass index), and Kruskal-Wallis H test for the skewed ones (i.e. years of schooling)

**Table 2.** Distribution of biomarkers characteristics of participants, with respect to the type of protein intake (Results are presented as mean±SD or P50 (P25-P75) (N=490)

Characteristics	Animal protein								Plant protein			
	Haem				Non-haem				Low	Moderate	High	p^†^
	Low	Moderate	High	p^†^	Low	Moderate	High	p^†^				
	n=164	n=163	n=163		n=164	n=163	n=163		n=164	n=163	n=163	
Urea, mg/dL	34 (29-42)	35 (30-43)	34 (29-41)	0.57	34 (29-41)	34 (29-42)	36 (30-41)	0.43	35 (29-42)	34 (30-43)	34 (29-40)	0.34
Uric acid, mg/dL	4.9±1.4	5.1±1.6	5.2±1.7	0.29	5.2±1.5	5.1±1.5	4.9±1.6	0.20	4.9±1.5	5.1±1.5	5.2±1.6	0.26
Creatinine, mg/dL	0.82±0.16	0.84±0.20	0.86±0.19	0.19	0.86±0.17	0.85±0.19	0.82±0.18	0.12	0.84±0.19	0.86±0.16	0.83±0.19	0.35
Total cholesterol, mg/dL	205±40	198±38	199±43	0.25	203±40	199±42	198±40	0.49	200±42	201±41	199±39	0.89
HDL-cholesterol, mg/dL	51±14	50±13	49±14	0.32	50±14	49±14	51±13	0.26	49±14	51±14	50±14	0.64
LDL-cholesterol, mg/dL	131±35	125±33	128±39	0.29	131±36	126±37	127±34	0.52	129±36	128±36	127±35	0.89
Triglycerides, mg/dL	100 (69-136)	99 (70-139)	88 (62-132)	0.19	97 (73-146)	102 (67-143)	81 (63-122)	0.02	92 (65-128)	93 (66-143)	100 (67-133)	0.72
Iron, μg/dL	89 (67-118)	91 (71-115)	85 (65-117)	0.86	97 (73-122)	88 (67-119)	83 (64-108)	0.01	86 (67-118)	90 (65-115)	90 (69-117)	0.91
C-reactive protein, mg/dL	0.10 (0.00-0.30)	0.10 (0.10-0.20)	0.10 (0.00-0.20)	0.09	0.10 (0.00-0.20)	0.10 (0.10-0.20)	0.10 (0.00-0.30)	0.97	0.10 (0.00-0.30)	0.10 (0.00-0.20)	0.10 (0.00-0.30)	0.57
Cystatin C, mg/L	0.87±0.17	0.85±0.16	0.81±0.15	<0.001	0.84±0.17	0.84±0.15	0.84±0.16	0.97	0.84±0.17	0.85±0.16	0.83±0.16	0.47
Haptoglobin, mg/dL	134±59	124±55	124±51	0.33	126±49	130±61	126±56	0.82	128±64	124±48	129±54	0.79
Ferritin, ng/dL	61 (31-102)	65 (30-123)	66 (32-142)	0.75	69 (36-139)	71 (32-116)	54 (28-99)	0.18	55 (28-108)	69 (32-129)	65 (36-125)	0.27
Haemoglobin, g/dL	13±1.3	13±1.3	14±1.4	<0.001	14±1.3	13±1.4	13±1.3	0.006	13±1.3	14±1.4	14±1.4	0.55
Haematocrit, %	40±3.6	41±3.7	42±4.1	<0.001	42±3.9	41±3.9	40±3.5	0.003	41±3.8	41±3.9	41±3.9	0.76
White blood cells, 10^3/μL	7.1±1.7	7.3±1.9	7.3±1.8	0.44	7.3±1.7	7.2±1.8	7.0±1.8	0.38	7.3±1.8	7.2±1.8	7.1±1.8	0.79
Monocytes, 10^3/μL	0.42±0.13	0.42±0.15	0.45±0.16	0.11	0.44±0.16	0.42±0.15	0.42±0.14	0.38	0.44±0.16	0.42±0.15	0.43±0.14	0.43
Platelets, 10^3/μL	234±51	232±53	229±55	0.65	227±53	237±50	231±56	0.28	241±56	230±55	224±47	0.02

^†^p values derived from one-way ANOVA for the normally-distributed variables (i.e. uric acid, creatinine, total cholesterol, HDL-cholesterol, LDL-cholesterol, cystatine C, haptoglobin, heamoglobin, haematocrit, white blood cells, monocytes, and platelets) and Kruskal-Wallis H test for the skewed ones (i.e. urea, triglycerides, iron, C-reactive protein, and ferritin)

However, residual confounding may exist; thus, the aforementioned analyses were repeated after taking into account the various potential confounders. Selected results from multi-adjusted linear regression models regarding the biomarkers that showed significance in the unadjusted analyses are presented in Table 3 (The other markers were also evaluated but the results are not presented here). Data analysis revealed that protein intake from haem animal origin was positively associated only with haemoglobin and haematocrit. Although protein intake from non-haem animal origin was negatively associated with iron, haemoglobin, and haematocrit, this relation was not evident in unadjusted analyses when age, sex, BMI, smoking, physical activity status, alcohol intake, and MedDietScore were additionally considered. Protein intake from plant origin was inversely associated only with platelets count but, again, this relationship was lost when additional adjustment for alcohol drinking and adherence to Mediterranean diet was taken into account. No other significant associations were observed between sources of protein intake and the investigated biomarkers (p>0.10 for all). Moreover, no significant gender-protein intake interactions on the levels of investigated biomarkers were observed; thus, the analyses were performed only on the entire sample to preserve statistical power.

## DISCUSSION

In this research work, the association between protein intake from haem and non-haem animals and that from plant origin and a series of haematological and biochemical markers relating to low-grade inflammation process was evaluated. Protein intake from haem animal origin was positively associated with haemoglobin and haematocrit levels while protein intake from non-haem animals and that from plants were not associated with any of the investigated biomarkers.

As reported above, protein intake from only haem animal origin was positively associated with haemoglobin and haematocrit levels whereas protein intake from non-haem animal origin and that from plant origin had no significant association on these haematological indices. This finding underlines the fact that not all sources of animal proteins are equivalent in their enhancing effects on iron absorption (21). It is likely that the facilitating effect of muscle tissue is more apparent with a more monotonous vegetarian diet that is typical in regions in which iron deficiency is prevalent (22). Moreover, other blood iron markers measured in this work (i.e. serum iron and serum ferritin levels) were not associated with the type of protein intake (i.e. haem, non-haem, and plant). There are conflicting results in the literature on this issue that have been mainly attributed to differences in blood losses (e.g. through menstrution) as well as various socioeconomic and behavioural determinants (e.g. smoking and physical activity), and dietary habits (23,24-28). By the exception of menstruation status, all the other potential confounders were taken into account in the present work.

Intake of protein from plant origin was inversely associated with platelets count but the relationship lost its significance when alcohol drinking and overall dietary habits (i.e. level of adherence to the Mediterranean diet) were taken into account (Model 4, Table 3). Thrombosis and inflammation are intricately linked because some of the same cellular and molecular players participate in both the processes. In particular, activated platelets express and secrete pro-inflammatory cytokines, which results in activation of monocyte integrins and in increased monocyte recruitment to the atherosclerotic lesions (29,30). A previous study has demonstrated that Mediterranean diet lowers blood platelet count in healthy subjects (31), a finding that could be attributed to the high content of antioxidant metabolites in plants that are abundant in Mediterranean diet and are known to have a platelet-lowering action, both *in vivo* and *in vitro* (32-34). Thus, the loss of significance of protein intake from plant origin on platelets count, observed in this work, may be due to the hidden effect of Mediterranean diet on platelets that overlapped the aforementioned association when entered in the model.

Finally, no significant association of the intake of non-haem animal protein with the investigated haematological and biochemical markers relating to inflammation was observed when characteristics of various participants were taken into account.

### Limitations

Despite the strengths of the presented results in terms of the variety of haematological and biochemical markers relating to low-grade chronic inflammation measured, the statistical power, and the various adjustments made that moderated residual confounding, this work had also some limitations. The most important limitation is the cross-sectional design which prohibited causal interpretations. Another limitation was that the observed findings were based on adults not suffering from diseases that might have influenced the levels of studied haematological and biochemical markers; thus, it is difficult to generalize the findings to people with health problems, such as thrombophilia. Moreover, although a validated FFQ was used, diet miss-reporting may always exist through this dietary evaluation method.

**Table 3.** Results from multiple linear regression models that evaluated the effect of type of protein intake on specific blood biomarkers (Results are presented as β-coefficients±SE, along with their 95% confidence intervals (CI) (n=490)

Parameter	Cystatin C, mg/dL		Haemoglobin, g/dL		Haematocrit, %	
	β±SE	95% CI	β±SE	95% CI	β±SE	95% CI
Model 1: Animal protein intake (haem), % of total energy	-1.44±0.15	(-0.010, 0.001)	0.09±0.02	(0.04, 0.13)	0.27±0.07	(0.14, 0.40)
Model 2: #1+Age and sex	-0.0002±0.003	(-0.005, 0.005)	0.04±0.02	(0.005, 0.08)	0.15±0.05	(0.04, 0.25)
Model 3: #2+BMI, smoking, and physical activity	-0.0007±0.003	(-0.006, 0.004)	0.04±0.02	(0.004, 0.08)	0.14±0.05	(0.04, 0.24)
Model 4: #3+Alcohol and MedDietScore	-0.001±0.003	(-0.006, 0.004)	0.04±0.02	(0.006, 0.08)	0.15±0.05	(0.04, 0.25)

	Triglycerides, mg/dL^†^		Iron, μg/dL^†^		Haemoglobin, g/dL		Haematocrit, %	
	β±SE	95% CI	β±SE	95% CI	β±SE	95% CI	β±SE	95% CI
Model 1: Animal protein intake (non-haem), % of total energy	-0.03±0.03	(-0.08, 0.02)	-0.05±0.02	(-0.09, -0.008)	-0.32±0.07	(-0.44, -0.19)	-0.93±0.18	(-1.3, -0.57)
Model 2: #1+Age and sex	-0.03±0.02	(-0.08, 0.02)	-0.03±0.02	(-0.07, 0.01)	-0.08±0.05	(-0.19, 0.02)	-0.27±0.15	(-0.57, 0.02)
Model 3: #2+BMI, smoking and physical activity	-0.02±0.02	(-0.06, 0.03)	-0.03±0.02	(-0.07, 0.01)	-0.07±0.05	(-0.18, 0.03)	-0.24±0.15	(-0.53, 0.05)
Model 4: #3+Alcohol and MedDietScore	-0.02±0.02	(-0.06, 0.03)	-0.02±0.02	(-0.06, 0.02)	-0.08±0.05	(-0.18, 0.03)	-0.25±0.15	(-0.55, 0.04)

	Platelets, 10^3/μL	
	β±SE	95% CI
Model 1: Plant protein intake, % of total energy	-12±4.0	(-20, -4.1)
Model 2: #1+Age and sex	-11±4.0	(-18, -2.9)
Model 3: #2+BMI, smoking and physical activity	-9.0±3.9	(-17, -1.3)
Model 4: #3+Alcohol and MedDietScore	-6.4±4.4	(-15, 2.2)

^†^Triglycerides and blood iron concentrations were log-transformed; Standard error

### Conclusions

Protein intake from only haem animal origin was associated with increased haemoglobin and haematocrit levels whereas protein intakes from non-haem animals and plants were not associated with the investigated haematological and biochemical markers of low-grade chronic inflammation when lifestyle factors and overall dietary habits were taken into account. These findings underline the need for future studies, like randomized clinical trials with dietary interventions, proposing different proportions of protein consumption from haem and non-haem animals and that from plants, and in various populations, to confirm or refute the presented results.

## ACKNOWLEDGEMENTS

Galenica SA and the Hellenic Heart Foundation funded the study (KA 00173). The authors are grateful to them and the men and women of Athens, who participated in and collaborated on this research. We also wish to express our gratitude to A. Giotopoulou, C. Katsagoni, E. Pagourtzi, K. Tsoutsoulopoulou, E. Serpanu, T. Rabbah, AV. Mitsopoulou (field investigators from Harokopio University and Polikliniki Hospital) for their substantial assistance in the enrollment of the participants.
